# Fine-scale genomic analysis of the tree endophyte *Annulohypoxylon* sp. FPYF3050 producing monoterpene 1,8-cineole

**DOI:** 10.1128/mra.01199-23

**Published:** 2024-09-25

**Authors:** Shuang Zhou, Guiming Dou, Dong-Hui Yan

**Affiliations:** 1 Key Laboratory of Forest Protection of National Forestry and Grassland Administration, Ecology and Nature Conservation Institute, Chinese Academy of Forestry, Beijing, China; University of California Riverside, Riverside, California, USA

**Keywords:** *Annulohypoxylon* genome, 1,8-cineole, nematicide, terpen-based advanced biofuel

## Abstract

1,8-Cineole has a potential in control crop pests and biofuels. The endophytic fungus, *Annulohypoxylon* sp. FPYF3050 (*Neolitsea pulchella*), can produce over 90% 1,8-cineole of relative area in its natural volatiles, possessed nematicide properties. The annotated genome of this strain will provide insights into potential application in biofumigation and terpene-based advanced biofuel.

## ANNOUNCEMENT

*Annulohypoxylon* sp. FYPF3050 was recovered from healthy branch bark of tree *Neolitsea pulchella* (1), growing in Hainan province, China, with surface sterilized in ethanol and chlorine bleach following Arnold's method (2). It is close to *A. stygium* and *A. astroroseum* based on phylogeny by markers ITS (KX578223), actin (KX592161), tubulin (KX592160), and EF-1 (KX708687) with all 100% confidences (1). Its volatile functioned nematicide against pine wilt nematode (3) and was dominant with 1,8-cineole more than 95% relative area in PDA medium and 91% relative area in poplar residues medium by GC-MS detection (1, 3). 1,8-Cineole is a monoterpene with multiply bioactivities, such as pest inhibition (3, 4), medicine (5), and an ideal additive for biofuel (1, 6). Therefore, annotating genome of this fungus could provide unique insight into potential applications in agricultural bio-fumigation and terpene-based advanced biofuel.

For genome sequencing, gDNA of the strain was extracted with a 2 × CTAB (cetyltrimethylammonium bromide) (2% CTAB mixing with beta-mercaptoethanol) buffer (7) after grounded mycelia in liquid N2, which harvested from potato dextrose agar plate growing 6 days at 25°C. The gDNA integrity, purity, and concentration were evaluated sequentially through 0.75% agarose gel, NanoDrop (Thermo), and Qubit 2.0 fluorimeter (Invitrogen). Covaris g-TUBE procedures sheared DNA 20 Kb fragments for SMRTbell Library. DNA size was selected using the BluePippin system (Sage) and loaded to sequencing with Magbead station. Six SMRT cells were executed the sequencing using P6-C4 chemistry in PaciBio RS II sequencer. A total of initial 486,649 reads were generated by the sequencing and resulted out of 377,276 reads of clean reads data through SMRTLink software using default parameters, with an average length of 9,766.41, a median length of 8,884.50, and N50 of 14,214 bp. Genome assembly was performed using the Hierarchical Genome Assembly Process (HGAP2.3.0) (8) with default settings. Long reads were selected as the seed sequences for genome pre-assembly using BLASR software (9) throughout comparison with other shorter reads using default parameters. The Celera assembler software was used for further assembly via the OLC algorithm with debug parameters (10). Finally, by comparing the original data to the assembled reference sequences, Quiver software was used to optimize the assembled results, correct assembly errors, and remove the low-depth contigs. Software Augustus (11), SNAP (12), and GeneMark-ES (13) were applied for *de novo* gene prediction from gene structures, and GeneWise was applied for gene homologues prediction (14). At last, EVidenceModeler gave the integrative results about gene prediction (15).

The strain's genome has an assembly size of 43.34 Mb with 13 contigs, contigs N50 of five, L50 of 3.9 Mbp, and no gaps. Sequencing read coverage depth is 85×. EvidenceModeler predicted 10,561 protein coding sequences, 33 rRNA, and 138 tRNA, with total length of 16,298,725 bp and average gene length of 1,543.3 bp. The antiSMASH 7.0 (16) presented 48 biosynthetic gene clusters within 10 cluster types, including seven BGC in two types related with terpenes. The information on the genome and BGC types, including data accession numbers, was summarized in Table 1 along with other strains in the genus (17).

**TABLE 1:** Information on sequenced genomes and biosynthetic gene clusters among *Annulohypoxylon* spp^*a*^

Strain		*A. bovei* CBS124037	*A. moriforme* CBS123579	*Annulohypoxylon* sp. FPYF3050
Genome sequencing information	genome size (Mbp)	41.76	42.66	43.34
	GC%	43.5	42.5	43.11
	contigs	571	1,005	13
	scaffold	496	967	**/**
	gene counts	11,346	11,828	10,561
	Protein-coding	11,202	11,683	10,561
	rRNA	**/**	**/**	33
	tRNA	**/**	**/**	138
	Bioproject accession	PRJNA567100	PRJNA567194	PRJNA1116787
	Biosamples accession	SAMN12794023	SAMN12794443	SAMN41574881
	SRA accession	SRS6016167	SRS6016086	SRR15113879
	Biosynthetic Gene Clusters -T1PKS + indole	/	/	1

^*a*^ “/” means no data provided.

## Data Availability

The strain is preserved in China General Microbiological Culture Collection Center (CGMCC), and the number is CGMCC 12771. The fine-scale project data are available under Genome assembly accession number ASM4010437v1 and BioProject accession number PRJNA1116787.
